# Association of glutathione S-transferases (*GSTT1*, *GSTM1* and *GSTP1*) genes polymorphisms with nonalcoholic fatty liver disease susceptibility: A PRISMA-compliant systematic review and meta-analysis

**DOI:** 10.1097/MD.0000000000030803

**Published:** 2022-09-23

**Authors:** Yi Zhu, Jian-Hua Yang, Jun-Ping Hu, Ming Qiao

**Affiliations:** a Department of Pharmacy, The First Affiliated Hospital of Xinjiang Medical University, Urumqi, China; b College of Pharmacy, Xinjiang Medical University, Urumqi, China.

**Keywords:** glutathione-S-transferase, meta-analysis, nonalcoholic fatty liver disease, polymorphism

## Abstract

Glutathione S-transferases (*GSTs*) genes single-nucleotide polymorphisms (SNPs) have been connected with the susceptibility of nonalcoholic fatty liver disease (NAFLD), but with inconsistent results across the current evidences. The present work was schemed to explore the association between *GSTs* genes polymorphisms and the NAFLD vulnerability via meta-analysis.

**Methods:**

PubMed, Web of Science, Cochrane Library, China National Knowledge Infrastructure and Wanfang were retrieved for eligible literatures previous to March 10, 2021. The odds ratio (OR) of the dichotomic variables and the standardized mean difference of quantitative variables with corresponding 95% confidence intervals (95%CIs) were computed to evaluate the strength of the associations. The quality of included studies were assessed via using Newcastle-Ottawa Scale (NOS).

**Results:**

In total, 7 case-control studies encompassing 804 NAFLD patients and 1362 disease-free controls in this meta-analysis. Ultimately, this analysis included 6, 5 and 5 studies for *GSTM1*, *GSTT1* and *GSTP1* polymorphisms, respectively. The pooled data revealed that the *GSTs* genes SNPs had conspicuous associations with NAFLD susceptibility: for *GSTM1*, null versus present, OR = 1.46, 95%CI 1.20 to 1.79, *P* = .0002; for *GSTT1*, null versus present, OR = 1.34, 95%CI 1.06 to 1.68, *P* = .01; for *GSTP1*, Ile/Val or Val/Val versus Ile/Ile, OR = 1.60, 95%CI 1.23 to 2.09, *P* = .0005.

**Conclusion:**

This work revealed that the *GSTM1* null, *GSTT1* null and *GSTP1*-Val genotypes might be related to increased NAFLD susceptibility.

## 1. Introduction

Nonalcoholic fatty liver disease (NAFLD) is gradually considered as the liver disease component of metabolic syndrome, which a risk factor for further development of fatty liver, nonalcoholic steatohepatitis (NASH), fibrosis, cirrhosis and hepatocellular carcinoma.^[1]^ According to the present data, NAFLD affects 10% to 30% of the general population in various countries, which it has been viewed as a huge global health burden.^[2]^ However, little is known about the latent mechanism involved in the development and pathogenesis of NAFLD. The onset of NASH on a background of fatty liver is believed to be due to an interplay between genetic and environmental factors, with a major role played by the oxidative stress.^[3]^ As the natural course of NAFLD and its progression to NASH is highly variable even with the same risk factors, it is reasonable that single nucleotide polymorphisms (SNPs) in genes potentially involved in oxidative stress could play a role in the disease onset and progression as reported by recent studies.^[4]^

Glutathione-S-transferases (*GSTs*) have a pivotal role as antioxidant defense mechanisms, and in conjunction with *SULT* and *CYP2E1*, products act by inactivating xenobiotics and products of oxidative stress.^[5]^

Lipid peroxides formed due to oxidative stress serve as endogenous substrates for *GSTs*. The *GSTs* are phase II metabolic enzymes and play a critical role in the defense against oxidative stress products and a variety of electrophilic compounds. Moreover, the *GSTs* family acts a significant role in antioxidant defence mechanisms via accelerating detoxification of electrophilic xenobiotics and deactivating a range of endogenous byproduct of oxidative stress.^[6-9]^ So far, studies have confirmed that *GST* enzymes in possession of 8 classes of soluble cytoplasmic isoforms, such as α-(A), ζ-(Z), θ-(T), κ-(K), μ-(M), π-(P), σ-(D), and ω-(O).^[10]^ In the last few years, *GSTT1*, *GSTM1*, and *GSTP1* have attracted much attention. Indeed, the *GSTT1*, *GSTM1*, and *GSTP1* genes encodes the θ, µ, π class of *GST* enzymes, and they are located on chromosomes 1p13.3, 22q11.2 and 11q13, respectively.^[11]^ In the human liver, hepatocytes contain high levels of *GSTM* and *GSTT*, whereas *GSTP* is predominantly expressed in the bile ducts.^[12]^ The *GSTM1* and *GSTT1* null genotypes are associated with deficiencies in *GSTM1* and *GSTT1* enzyme activity and the *GSTP1*-Val (105) polymorphism is associated with altered catalytic function. Individuals with *GSTM1* and *GSTT1* null genotypes or *GSTP1*-Val (105) would be expected to have decreased *GST* detoxification and, thus, potential increases in the levels of toxic metabolites.^[13]^ At present, genome wide association studies have demonstrated that *GSTs* had been verified the crutial role in the disease onset and progression of NAFLD.

A limited number of studies have evaluated the association of *GST* genotype profile with liver-related diseases. Several have reported a positive association between the *GSTM1* and *GSTT1* null genotypes and *GSTP1*-Val (105) polymorphisms and increased risk of alcoholic pancreatitis, alcoholic cirrhosis, hepatocellular carcinoma, hepatis B virus infection progression, NAFLD, and cryptogenic cirrhosis development. It was reported that *GSTT1*/*GSTM1* deletions (*GSTT1*/*GSTM1* null) could inhibit detoxification of *GSTT1*/*GSTM1* substrates that were either toxicant or carcinogen. Double null genotypes of *GSTM1* and *GSTT1* might give rise to a complete lack of enzymatic activity.^[14]^ Moreover, *GSTM1* and *GSTT1* null genotypes were associated with type 2 diabetes and its complications which were closely related to NAFLD with several reports underlining the role of *GSTT* on liver damage.^[15-17]^ Furthermore, the *GSTP1* gene polymorphism was the outcome of a single nucleotide substitution of A to G, which leaded to valine instead of isoleucine in the binding site of *GSTP1* and altered catalytic activity of enzyme.^[18, 19]^ While the *GSTP1*-Val (105) variant genotype has also been associated with an increased risk for advanced liver diseases of various causes.^[20]^

Several studies have appraised the relationship of *GST*s genes SNPs and liver-related diseases. It indicated that the null genotypes of *GSTM1*/*GSTT1* and *GSTP1*-Val (105) genes SNPs were related to the risk of NAFLD.^[21-27]^ However, these studies yielded varying and divergent results. Accordingly, a meta-analysis was carried out to supply a more accurate and synthetic assessment on the relationship of *GSTM1*, *GSTT1*, *GSTP1* genes polymorphisms and the NAFLD susceptibility.

## 2. Materials and Methods

### 2.1. Literature collection

Two independent researchers searched the PubMed, Web of Science, Cochrane Library, China National Knowledge Infrastructure and Wanfang databases prior to March 10, 2021. The searching strategy of PubMed was exhibited as follows: (“nonalcoholic fatty liver disease [Mesh]” OR “nonalcoholic fatty liver disease” OR “NAFLD” OR “nonalcoholic steatohepatitis” OR “NASH”) AND (“*GSTT1*” OR “*GSTM1*” OR “*GSTP1*” OR “glutathione S-transferase” [Mesh]) AND (Single Nucleotide Polymorphism[Mesh] OR Variant OR SNP OR Polymorphism OR mutant OR mutation OR variation). No language restriction was set. This study depended on previously published literature and public databases. Thus, this work does not need ethical approval and patient consent.

### 2.2. Inclusion and exclusion criteria

Original studies were incorporated into this analysis in the light of the following inclusion criteria: case-control studies; study investigated the associations of *GSTs* polymorphisms and NAFLD predisposition; NAFLD is diagnosed by pathology or ultrasound; control subjects were disease-free individuals; detailed genotype data can be calculated for odds ratios (ORs) and 95% confidence intervals (95% CIs). Correspondingly, reviews, conference abstracts, commentary articles, letters to editor, animal studies, unpublished data, case reports, as well as family-based studies were excluded.

### 2.3. Methodological quality assessment

Two investigators evaluated the study quality based upon Newcastle-Ottawa Scale (NOS).^[28]^ The NOS is composed of 3 aspects, namely selection, comparability and exposure. Each study could receive 0 to 9 scores. Nevertheless, studies with ≥ 6 scores were regarded as high-quality studies. Disagreement was resolved by discussion.

### 2.4. Data extraction

The extracted information contained the name of first author, year of publication, country, ethnicity and gender of enrolled subjects, numbers of NAFLD and control subjects, diagnostic methods of NAFLD, genotyping of enrolled subjects and Hardy-Weinberg equilibrium (HWE) results.

### 2.5. Statistical analysis

The control participants of incorporated studies were estimated via HWE.^[29]^ Summary ORs with 95% CIs were computed to specifically evaluate the relationship of *GSTs* polymorphisms and the NAFLD susceptibility. Cochran’s *Q* test and *I*^2^ test were applied to appraise the between-study heterogeneity.^[30]^ The random-effect model was employed to calculate merged ORs if *P* < .1, *I^2^* > 50%. If not, the fixed-effect model was utilized for data synthesis.^[31]^ Sensitivity analysis was performed as well to assess the stability of all incorporated studies via the leave-one-out method. When the included studies were more than 10, funnel plot was employed to valuated the publication bias.^[32]^ On the contrary, less than 10 studies are not required. All analyses were done using RevMan 5.3 software.

## 3. Results

### 3.1. Literature search

**Figure 1.**
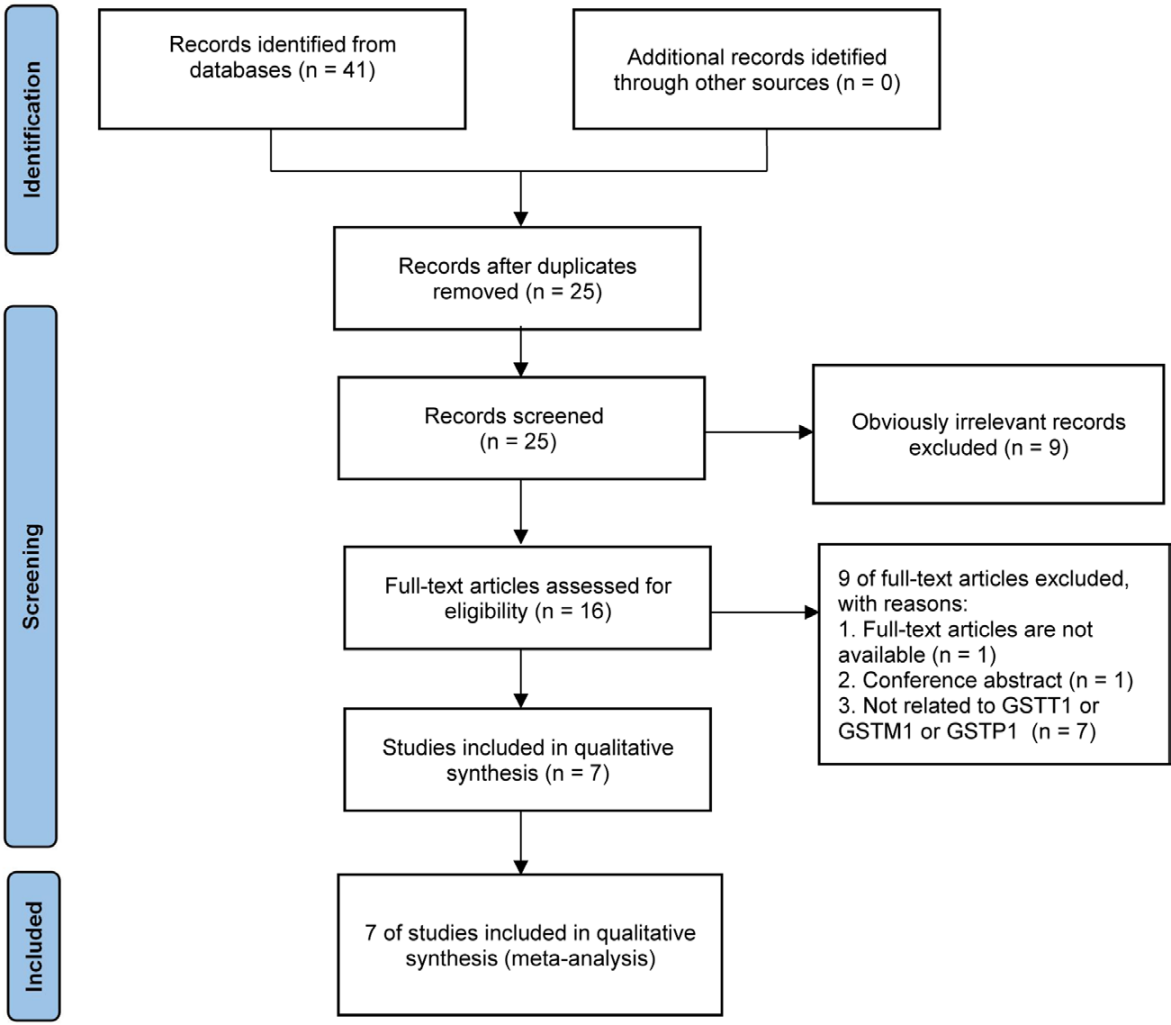
Flow chart of literature search and screen.

Primary search of electronic databases retrieved 41 potentially relevant publications: 17 from PubMed, 20 from Web of Science, 0 from Cochrane library, 1 from China National Knowledge Infrastructure and 3 from Wanfang. No additional records were acquired from other sources. And then 25 studies remained after removing duplicated articles. Subsequently, a total of 9 irrelevant articles were excluded on the basis of titles and abstracts screening. After applying inclusion and exclusion criteria, 7 unrelated articles, 1 conference abstract were removed and 1 full-text article was not available. Ultimately, 7 studies went into the process of meta-analysis. Overall, a flowchart summarizing the procedure of literature identification was illustrated in Figure 1.

### 3.2. Main characteristics

The studies were performed in Italy, Japanese, Iran and Ukraine. Four studies were conducted in the Asian population and 3 studies were in the Caucasian population. The diagnostic method for NAFLD in these studies was ultrasonography. In total, 7 studies encompassing 804 case and 1362 control participants were analyzed in current analysis. For *GSTM1*, *GSTT1* and *GSTP1* genes SNPs, there were 6, 5 and 5 studies ultimately incorporated, respectively. They were all case-control designed and published between 2008 and 2020 (Table 1). In addition, studies with ≥ 6 scores were regarded as high-quality studies according to evaluation of methodological quality (Table 2).

**Table 1:** Main characteristics of the included studies.

							Case		Control		
Study	Yr	Country	Ethnicity	Gender	Means of diagnosis	Sample size	Null	Present	Null	Present	HWE
***GSTM1***											
Luca M	2014	Italy	Caucasian	both	Ultrasonography	234/349	145	147	187	172	0.54
Masaharu H	2008	Japanese	Asian	both	Ultrasonography	69/184	40	29	84	100	<0.05
Mohammad H	2012	Iran	Asian	both	NA	83/93	48	35	36	57	0.015
Kentaro O	2013	Japanese	Asian	both	Ultrasonography	130/566	74	56	277	289	0.12
Tamandani D	2011	Iran	Asian	NA	NA	80/80	11	69	7	73	0.3
Vasyl P	2020	Ukraine	Caucasian	both	Ultrasonography	104/45	52	52	23	22	NA
***GSTT1***											
Luca M	2014	Italy	Caucasian	both	Ultrasonography	234/349	75	217	83	276	0.45
Masaharu H	2008	Japanese	Asian	both	Ultrasonography	69/184	37	32	85	99	0.09
Mohammad H	2012	Iran	Asian	both	NA	83/93	2	81	0	93	0.221
Kentaro O	2013	Japanese	Asian	both	Ultrasonography	130/566	61	69	249	317	0.49
Vasyl P	2020	Ukraine	Caucasian	both	Ultrasonography	104/45	18	86	6	39	NA
***GSTP1***							Ile/Ile	Ile/Val or Val/Val	Ile/Ile	Ile/Val or Val/Val	
Masaharu H	2008	Japanese	Asian	both	Ultrasonography	69/184	49	20	142	42	0.14
Mohammad H	2012	Iran	Asian	both	NA	83/93	29	54	53	40	0.003
Kentaro O	2013	Japanese	Asian	both	Ultrasonography	130/566	89	41	424	142	0.13
Tamandani D	2011	Iran	Asian	NA	NA	80/80	9	71	10	70	0.1
Prysyazhnyuk VP	2017	Ukraine	Caucasian	NA	NA	104/45	47	57	28	17	NA

**Table 2:** Quality assessment of included studies based upon the Newcastle-Ottawa Scale (NOS).

Item/Study	Luca M	Masaharu H	Mohammad H	Kentaro O	Tamandani D	Vasyl P	Prysyazhnyuk VP
	2014	2008	2012	2013	2011	2020	2017
**Selection**							
Adequate definition of cases	1	1	1	1	1	1	1
Representativeness of cases	1	1	1	1	1	0	0
Selection of control subjects	1	0	0	0	0	1	0
Definition of control subjects	1	1	1	1	1	1	1
**Comparability**							
Control for important factor or additional factor	1	1	1	1	1	2	1
**Exposure**							
Exposure assessment	1	1	1	1	1	1	1
Same method of ascertainment for all subjects	1	1	1	1	1	1	1
Non-response rate	1	1	1	1	1	1	1
Total score	8	7	7	7	7	8	6

### 3.3. GSTM1 gene polymorphism and NAFLD susceptibility

**Figure 2.**
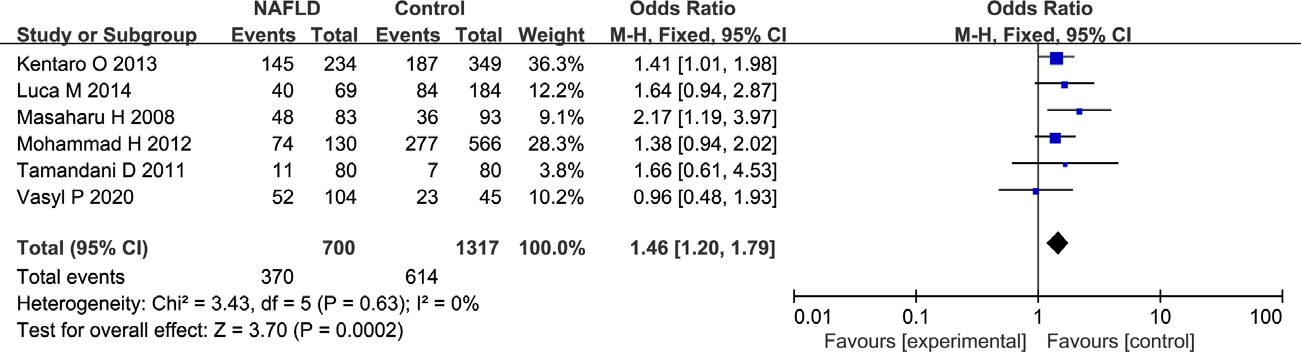
Effect of the *GSTM1* null versus present genotype on the risk of NAFLD. NAFLD = nonalcoholic fatty liver disease.

In total of 6 studies including 700 NAFLD patients and 1317 controls for *GSTM1* gene polymorphism. The fixed-effects model was employed for data analysis on account of the heterogeneity in between-study was not remarkable. It revealed that *GSTM1* was appreciably connected with the NAFLD vulnerability (null vs present, OR = 1.46, 95%CI: 1.20–1.79, *P* = .0002; Fig. 2).

### 3.4. GSTT1 gene polymorphism and NAFLD susceptibility

**Figure 3.**
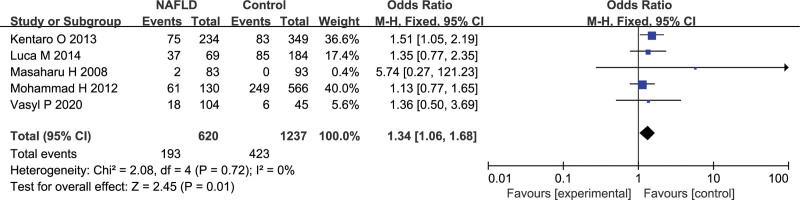
Effect of the *GSTT1* null versus present genotype on the risk of NAFLD. NAFLD = nonalcoholic fatty liver disease.

Overall, 5 researches containing 620 NAFLD and 1237 healthy subjects for *GSTT1* to perform data analysis. There was no heterogeneity amidst studies for *GSTT1* (*P* = .72, *I^2^* = 0%). So the fixed-effects model was performed for data analysis. The pooled data indicated there was a noticeable association between the SNP of *GSTT1* and the NAFLD susceptibility (null vs present, OR = 1.34, 95%CI: 1.06–1.68, *P* = .01; Fig. 3).

### 3.5. GSTP1 gene polymorphism and NAFLD susceptibility

**Figure 4.**
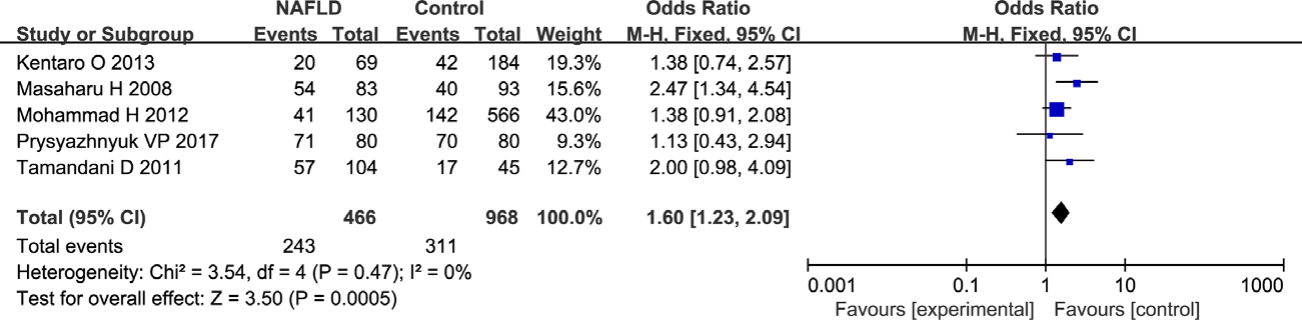
Effect of the *GSTP1*-Val versus *GSTP1*-Ile allele on the risk of NAFLD. NAFLD = nonalcoholic fatty liver disease.

In total of 5 studies with 466 cases and 968 controls were used for data pooled. The fixed-effect model was carried out to estimate the association of *GSTP1* gene polymorphism and the NAFLD risk by virtue of no heterogeneity among studies (*P* = .47, *I^2^* = 0%). The results indicated a obvious association between *GSTP1* gene polymorphism and NAFLD susceptibility (Ile/Val or Val/Val vs Ile/Ile, OR = 1.60, 95%CI 1.23–2.09, *P* = .0005; Fig. 4).

### 3.6. Sensitivity analysis and publication bias

After the omission of an individual study, the recalculated *P* value, ORs and 95%CIs did not change substantially. Therefore, the outcomes were considered to be statistically robust and reliable. The funnel plot for assessment of publication bias was not implemented on account of less than 10 researches.

## 4. Discussion

Despite the specific pathological mechanism of NAFLD still needs to be explored. Nevertheless, research increasingly revealed that genetic predisposition plays an crucial intrinsic role in the occurrence and development of NAFLD. In addition, SNPs in human might be one of the critical steps to disclose the genetic factor for NAFLD pathogenesis. With further research, *GSTs* genes as a genetic factor have obtained increasing attention over current years. So far, the present researches have been implemented concerning the relationship of *GSTM1*, *GSTT1* and *GSTP1* genes polymorphism and the NAFLD vulnerability with inconsistent conclusions. This inconsistency might be caused by factors like limited sample sizes, confounding factors, as well as clinical heterogeneity of NAFLD. Therefore, we collected the existing evidence and looked into the associations of *GSTs* genes SNPs and the NAFLD vulnerability via meta-analysis, which could combine data from individual studies, examine and explain the heterogeneity, and increase the statistical power. In conclusion, the merged data suggested a significant correlation between *GSTM1*, *GSTT1* and *GSTP1* genes SNPs and the NAFLD vulnerability. Of note, the recalculated *P*-value, ORs and 95%CIs did not change substantially after the omission of an individual study. In this work, it demonstrated that the frequency of *GSTM1* null, *GSTT1* null and *GSTP1*-Val allele genotypes in NAFLD patients was remarkably higher than that in healthy subjects.

*GSTs* are enzymes in the second-stage detoxification system, which can not only catalyze reduced glutathione sulfhydryl groups, but also neutralize lipid and DNA oxidation products, and have protective effects against endogenous oxidative stress and exogenous toxins.^[33,34]^ *GSTs* is distributed in cytosol, mitochondria and microsome. *GSTs* abnormalities are associated with many diseases, such as malignancies, neurodegenerative diseases, for example, parkinsonism, immune diseases e.g. diabetes and asthma. Hence, almost half the population had *GST* mutations and was susceptible to whichever toxin-induced disease. Among them, *GSTT1*, *GSTM1* and *GSTP1* have garnered considerable attention from various research teams around the world in the recent decade.^[35]^ Several investigations have disclosed that homozygous deletion of *GSTM1* and *GSTT1* genes (*GSTM1* null and *GSTT1* null) were connected with lack of relevant *GST* isoenzyme synthesis and augmented the susceptibility of genetic damage.^[36,37]^ Furthermore, the double null genotypes of *GSTT1* and *GSTM1* genes could decline the activity of sulfhydryl binding so as to induce insufficient activity of detoxification in the body.^[38,39]^ *GSTP1* gene polymorphism was the outcome of a single nucleotide substitution of A to G, which leaded to valine instead of isoleucine in the binding site of *GSTP1* and alters catalytic activity of enzyme.^[40,41]^ Rossini et al found the opposite role for *GSTP1* and *GSTT1* polymorphisms in the risk of esophageal squamous cell carcinoma.^[42]^ Yet, other studies have reported a protective effect of the *GSTT1* null genotype for different tumors, such as of the lung, breast and bladder.^[43]^ In addition, those who have the *GSTT1* null genotype were more resistant to DNA damage caused by polycyclic aromatic hydrocarbons compared with wild-type *GSTT1* individuals.^[44]^ Wu et al reported that the 313th G/G polymorphic variation of the *GSTP1* gene was one of the risk factors for the emergence of the urinary bladder cancer.^[45]^ Previous reports also suggested that *GSTM1/GSTT1* null or *GSTP1*-Val genotypes were remarkably associated with the vulnerability of hepatis B virus, hepatocellular carcinoma, alcoholic cirrhosis, and NAFLD.^[46-51]^ Moreover, the *GSTM1* null genotype was reported to be more common in NAFLD patients than in controls, and *GSTP1*-Val was proved to be a hazard for NAFLD vulnerability in the Iranian population.^[52]^

*GSTs* mutations were seen as a host susceptibility factor that works only in presence of a specific type of toxin and its amount. Zaki et al reported that 50% of Egyptian children with diabetes have almost 50% deletions of *GSTM1*, which was comparable to the general population, yet an unforeseen factor seemed to make these children develop the diabetes type 2 disease.^[53]^ In the same study, the children with type 2 diabetes had other detoxification defects as *GSTT1* and *GSTP1*. Hence development of a disease phenotype might prove to be the result of exposure to a specific chemical in an amount that cannot be handled by the dysfunction of detoxification of the host. Consequently, it proved that detoxification defects that confered host susceptibility, but were not the sole determinant factor of disease pathogenesis, elinical picture, march or outcome.^[54]^

Up to now, this is the first synthetical study on the relationship between *GSTs* polymorphisms and NAFLD vulnerability. There were several strengths in this study. First, to gather a maximum amount of relevant literature, a comprehensive search strategy was adopted to retrieve eligible studies in both English and Chinese databases. Besides, the methodological quality of studies was evaluated via NOS, which allowed for the judgment of potential risk of bias. According to the NOS, all eligible studies were of high methodological quality. Furthermore, sensitivity analyses were carried out in this study, which guaranteed the reliability of the findings.

Nevertheless, there still existed several drawbacks should be acknowledged. First, only 7 studies were included, the statistical power was limited, and subgroup analyses were not carried out because of the limited degree of freedom. Second, the absence of HWE in individual studies may lead to information bias. Third, we ignored the synergistic effect of polymorphism at other sites of NAFLD because only 3 loci in the *GST* gene were studied in association with susceptibility to NAFLD. Thus, interactions between these loci and genes may result in concealing or amplifying the actual function of individual loci or genes. Leave aside these drawbacks, this study is the first to provide a more accurate and powerful evidence on the association between *GSTM1*, *GSTT1* and *GSTP1* genes polymorphisms and NAFLD vulnerability.

## 5. Conclusion

In brief, it revealed that *GSTM1* null, *GSTT1* null and *GSTP1*-Val genotypes were appreciably associated with augmented risk of NAFLD vulnerability. Concerning limitations of this study, it is necessary to confirm the present findings by complementary studies with larger sample size.

## Acknowledgments

Authors would like to thank all studies and their participants involved in this investigation.

## Author contributions

**Conceptualization:** Yi Zhu.

**Supervision:** Ming Qiao.

**Validation:** Jianhua Yang, Junping Hu.

**Writing – original draft:** Yi Zhu.

**Writing – review & editing:** Ming Qiao, Jun-Ping Hu.
